# Utility of Faster R-CNN in methodological comparison and evaluation of reticulocytes

**DOI:** 10.3389/fphys.2024.1373103

**Published:** 2024-05-31

**Authors:** Shengli Sun, Geng Wang, Binyao Zhang, Fei Wang, Wei Wu

**Affiliations:** Department of Clinical Laboratory, Peking Union Medical College Hospital, Beijing, China

**Keywords:** intelligent learning system, methodological comparison, reticulocyte, clinical laboratory, hematology analyzers

## Abstract

**Objective:**

The purpose of this study was to evaluate the methodological comparison of reticulocytes by using the intelligent learning system Faster R-CNN, a set of reticulocyte image detection systems developed using deep neural networks.

**Methods:**

We selected 59 EDTA-K2 anticoagulated whole blood samples and calculated the RET% using seven different Sysmex XN full-automatic hematology analyzers with Faster R-CNN in the laboratory. We compared and evaluated the methods and statistically analyzed the correlation between the various test results.

**Results:**

The results indicated a high degree of consistency between the seven Sysmex XN full-automatic hematology analyzers and Faster R-CNN in detecting RET%. The correlation coefficients were 0.987, 0.984, 0.986, 0.987, 0.987, 0.988, and 0.986, respectively.

**Conclusion:**

We found that the Sysmex XN full-automatic hematology analyzers in our laboratory using the Faster R-CNN system met the requirements of the methodological comparison of reticulocyte detection and this intelligent learning system can be a useful clinical tool.

## 1 Introduction

Faster R-CNN is a software that takes a microscope image as input and outputs cell information including location and actual class. Faster R-CNN deep neural network to train a reticulocyte (RET) detection model, which shows outstanding performance including high accuracy and fast speed. Both the recall and precision rate of the model are more than 97%, and average analysis time of a single image is 0.21 s. Previous evidence has revealed that the deep learning method holds the potential to act as a rapid computer-aid tool for manual RET enumeration for cytological examiners and might further increase its performance when combined with automated slide scanner (Wang et al., 2021).

RET are the immature red blood cells between late immature red blood cells and mature red blood cells. They reflect the erythropoiesis activity of human bone marrow and the hematopoietic function of the red blood cell system (Peng et al., 2018). The two current methods to detect RET in the laboratory are the traditional manual microscope examination and the automatic blood cell analyzer. Automatic RET detection technology is gradually replacing traditional manual microscopy examination due to the growing demand for rapid clinical diagnosis and treatment (Li et al., 2014). However, manual microscopy examination remains the “gold standard” of RET counting, which is used to evaluate the performance of instruments and diagnose blood system diseases (Bain, 2005).

Manual microscopic examination of RET varies from person to person, is easily influenced by subjective factors, and has poor repeatability. The accuracy of the results can vary with the number of cells counted, the thickness of the blood smear, the effect of ribonucleic acid (RNA) staining, and the temperature and time of staining (Wang, 2010). Computer vision technology-assisted medical image analysis of blood smear microscopy has actively improved the automation level of microscopic examination (Mohammed et al., 2014; Da Costa, 2015).

In this study, we evaluated the deviation between the Faster R-CNN counting RET and the RET detected by seven hematology analyzers from the same manufacturer, aiming to find a method with strong repeatability and relatively convenience for detecting RET%.

## 2 Materials and methods

### 2.1 Sample collection

In September 2022, we collected venous blood samples from 59 outpatients and inpatients of the Peking Union Medical College Hospital (PUMCH). We collected 2 mL of venous blood from each patient with EDTA-K2 anticoagulant using vacuum blood collection tubes (Becton, Dickinson and Company, United States). The samples were stored at room temperature and tested within 4 h of collection. The 59 samples were respectively 5 samples (A, B, C, D, and E) for personnel parallel experiment, 49 samples for evaluation of the Faster R-CNN system and Sysmex XN, 5 samples with RET% < 1.0 for the Faster R-CNN system counting 1,000 and 2,000 red blood cell.

### 2.2 Sysmex XN

Sysmex XN full-automated hematology analyzer and matching reagents, calibrators, and high, medium, and low concentration quality control products; In Fluorocell RET, we used a fluorescent dye to stain the reticulocyte nucleic acid treated with CELLPACK DFL. The RET percentage (RET%), the absolute value of RET (RET#), immature RET percentage (IRF%), low fluorescence RET ratio (MFR%), and medium fluorescence RET ratio (RET%) were obtained using the lateral fluorescence (X-axis) and forward scattered light signals (Y-axis), which were detected using semiconductor laser flow cytometry. In strict accordance with the Standard Operating Procedure (SOP) of the machine, the machine was calibrated (including RET parameters) with the supporting calibrator XN-L CHECK every 6 months in the automated hematology analyzer. High, medium, and low concentration quality control products of XN Sysmex Company were tested every day in the fully automated hematology analyzer. The results of the different quality controls were uploaded to Sysmex Network Communication Systems (SNCS—the global laboratory inter-room quality control model for calculating the daily quality control of laboratories), and we ensured that all the controls were qualified. Seven analyzers were used to ensure the stability of the detection results in our laboratory.

### 2.3 Faster R-CNN

Faster R-CNN is a software used by laboratory technicians to capture images and automatically count RET% after staining with brilliant cresyl blue. Three technicians were assigned to detect differences among five samples, primarily to eliminate variations in detecting the same sample among different personnel and ensure the accuracy of the test results. Following the SOP guidelines, the same blood smear stained with brilliant cresyl blue was examined by three technicians using Faster R-CNN to count RET%. The microscope we use is Olympus CX31, and the camera is Basler acA 1920. The computer hardware for deep learning is Intel i9- 9900K CPU, 32GB RAM, and Nvidia RTX 2080Ti GPU. The software applied is Ubuntu 18.04, Pytorch 1.3, and Python 3.5.

### 2.4 Methods

Following the WS/T 346-2011 “Reference Method for Reticulocyte Counting” (Health industry standard of the People’s Republic of China, 2012), one drop of brilliant cresyl blue physiological saline solution was placed in a small test tube, and one drop of the blood sample was added and mixed well. It was allowed to stand for 15–20 min to prepare the blood smear. We used red blood cells that were uniformly distributed. With good staining, Faster R-CNN can calculate 2,000 red blood cells. The same time 59 samples were immediately detected in the Sysmex XN full-automated hematology analyzer. Five samples (A, B, C, D, and E) for personnel parallel experiment, 49 samples for evaluation of the Faster R-CNN system and Sysmex XN, 5 samples with RET% < 1.0 for the Faster R-CNN system counting 1,000 and 2,000 red blood cell.

## 3 Data analysis

We used SPSS 19.0 software for statistical analysis. Paired t-test was used to compare the correlation between the two groups of data, and the difference was statistically significant at *p* < 0.05. Calculation formula: coefficient of variation (CV%) = standard deviation/mean *100, Deviation% = (instrument test result - personnel test result)/personnel test result *100%.

## 4 Results

### 4.1 Faster R-CNN personnel count RET comparison

Three qualified morphology instructors (1, 2, and 3) used Faster R-CNN to count the five samples (A, B, C, D, and E) and the CV% of each sample of the three instructors was less than 15% (1/2 TE), as shown in Table 1. The coefficient of variation of RET% among different instructors using the Faster R-CNN statistics was within the acceptable range.

**TABLE 1:** Comparison of RET% counting results from three instructors using faster R-CNN.

	A	B	C	D	E
1	15.8	1.0	9.4	1.4	7.0
2	16.2	1.1	9.1	1.8	7.6
3	16.0	1.0	9.4	1.5	7.1
SD	0.20	0.06	0.17	0.21	0.32
Mean	16.00	1.03	9.30	1.57	7.23
CV%	1.25	5.59	1.86	13.29	4.44

### 4.2 Correlation between the RET% counting of Faster R-CNN and the machine

As shown in Table 2, the correlation between the RET% detected using the seven Sysmex XN full-automated hematology analyzers and the RET% counted using FASTER R-CNN was greater than 0.98, indicating a good correlation (*p* < 0.01).

**TABLE 2:** Comparison of RET% detected using Sysmex XN and Faster R-CNN.

	Faster R-CNN	8	7	6	5	Emergency instrument 4	Emergency instrument 3	Emergency instrument 2
Minimum	0.40	0.40	0.43	0.37	0.33	0.28	0.34	0.34
Maximum	36.00	33.63	33.36	31.23	32.07	33.87	30.71	33.87
Mean	5.80	5.69	5.68	5.55	5.55	5.67	5.34	5.73
Correlation	1.000	0.987	0.986	0.984	0.987	0.986	0.988	0.987
Significance	<0.01	<0.01	<0.01	<0.01	<0.01	<0.01	<0.01	<0.01

**FIGURE 1:**
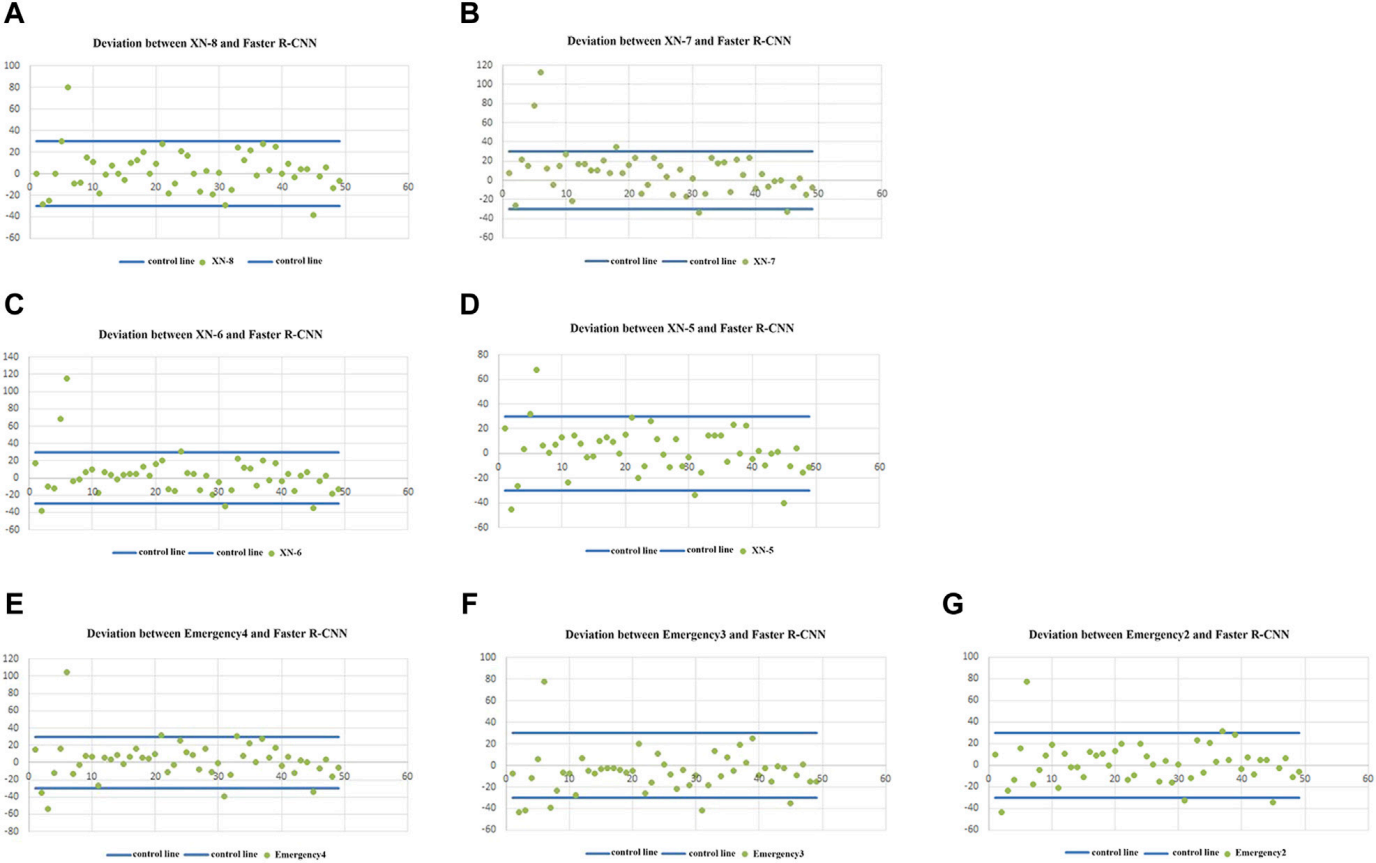
**(A–G)** show the scatter distribution of the difference between the RET% detected using the seven Sysmex XN full-automated hematology analyzers (8, 7, 6, 5, emergency-4, emergency-3, and emergency-2), respectively, and the RET% counted using Faster R-CNN.

The scatter plots of the deviation between the RET% detected using Sysmex XN and the RET% counted using Faster R-CNN in 49 samples are shown in Figure 1, respectively. The deviation between the RET% detected using XN and the RET% counted using Faster R-CNN was within the acceptable deviation range of over 90%, but we found that the deviation between the RET% detected using XN and the RET% counted using Faster R-CNN was relatively large when RET% < 1.0%.

### 4.3 Comparison of counting different numbers of red blood cells using Faster R-CNN in low value RET

Differences in the results of the RET counting of 5 samples (RET% < 1.0) with Sysmex XN-8 detection (A), counting 1,000 red blood cells by Faster R-CNN (B), and counting 2000 red blood cells by Faster R-CNN (C) are shown in Table 3.

**TABLE 3:** Comparison between XN-8 and Faster R-CNN when RET% < 1.0

Sample	A	B	C	Deviation between A and B (%)	Deviation between C and A (%)
1	0.88	0.8	0.7	−10.0	−25.7
2	0.69	0.5	0.6	−38.0	−15.0
3	0.61	0.7	0.5	12.9	−22.0
4	0.84	1.0	0.8	16.0	−5.0
5	0.88	0.8	0.9	−10.0	2.2

## 5 Discussion

We compared the accuracy of the RET% test results of seven Sysmex XN full-automated hematology analyzer tests in the laboratory (namely, Sysmex Emergency instrument-2, Sysmex Emergency instrument-3, Sysmex Emergency instrument-4, Sysmex XN-5, Sysmex XN-6, Sysmex XN-7, and Sysmex XN-8 for target machines in the laboratory), We found that the Sysmex XN full-automatic hematology analyzers in our laboratory using the Faster R-CNN system met the requirements of the methodological comparison of reticulocyte detection, The Faster R-CNN system can be a useful clinical tool.

Classifying and counting whole blood cells are the most commonly used testing methods in hematology laboratories. With an increase in the number of laboratory samples and the in-depth study of intractable diseases, automated hematology analyzers provide more accurate results due to their constantly evolving and updated technologies (International Council for Standardization in Haematology et al., 2014). The intelligentized coordination between the modular blood analysis and the System XN blood analysis systems enable efficient detection of a large number of samples (Liao et al., 2016). RET is an immature red blood cell that reflects the hematopoietic function of erythroid bone marrow and is an important index to diagnose the effectiveness of treatment for anemia and related diseases (Luan et al., 2011; Wu and Zhang, 2012; Gu and Wei, 2013; Wang et al., 2013; Zhang and Ding, 2016). The new parameters of RET are gradually being used in clinical settings and have proved helpful to distinguish the types of anemia and evaluate the hematopoietic function of patients after chemotherapy and bone marrow transplantation, while also being highly valuable in clinical disease diagnosis (Liu, 2018).

Intelligent medicine allows for more objective diagnostic outcomes for clinical diagnosticians with the advancement of imaging and deep learning technologies. As the establishment of a training model requires a lot of data, the more data there is, the more obvious the advantages of deep learning and the lower the error rate. Wang et al. (2021) used the Faster R-CNN system image amplification technology to identify RET% with a false positive rate of 0.6% and a false negative rate of 2.7%. The accuracy of red blood cell recognition was over 97%, and the recognition time of a single red blood cell was 0.01 s. Compared with the traditional manual microscopic examination using an abalone counting board, the Faster R-CNN analysis system provides better traceability, and the images captured using Faster R-CNN can be preserved permanently. When the results are disputed, a re-analysis of the results can be done based on the original photographs. While the manual microscope is still the “gold standard” for evaluating the performance of instruments, Sysmex XN has excellent traceability in the calibration of RET testing items (Bain, 2005). Faster R-CNN not only realizes the manual microscope traceability of RET counting but also allows for longer-term storage of results. In an earlier study in our laboratory, we found that the number of red blood cells detected and analyzed using the Faster R-CNN analysis system was more than 2,000, which was more accurate than using the manual microscope (Wang et al., 2021).

The WS/T 346-2011 reference method for reticulocyte count (Health industry standard of the People’s Republic of China, 2012) recommends that the specimen be stained within 4 h. To eliminate any potential time-related bias, we used the Sysmex XN automated hematology analyzer to detect RET% immediately after bright cresyl blue staining, and 15–20 min following that, Faster R-CNN was used to count RET%. We found a good correlation between the RET% counted using Faster R-CNN and the RET% detected using the automated hematology analyzer. The comparative analysis of the counting results of the entire single sample showed that the instrumental method was slightly higher than the Faster R-CNN, especially with the decrease of RET%, and the deviation increased obviously, which is consistent with the research results of Li et al. (2014). However, three samples had a large deviation (50%) among the seven Sysmex XN comparison results. The instrument detection results of RET% and Faster R-CNN RET% were sample 5 (0.7; 0.4), sample 18 (18.97; 30.90), and sample 31 (0.43; 0.6).

In sample 18, the instrument testing showed lower counting results. We found that there were still some scattered points outside the RET detection gate, which led to the low detection result of RET% in sample 18, but this phenomenon was not found in the other two samples with more RETs (samples 46 and 47). Samples 5 and 31 showed a large deviation from the RET% of Faster R-CNN. This can be explained by the possibility of massive red blood cells counted as RET in the instrumental method through flow cytometry, reflecting the discrepancy between RET% detected using the instrumental method and RET% counted using Faster-CNN when the RET is less in quantity. Therefore, we selected five patients with RET% < 1.0 and compared the total number of 1,000 cells and 2,000 cells using Faster CNN and found that there was no change in the deviation trend. This indicated that when there is a small number of RET, increasing the total number of cells in Faster R-CNN did not change the discrepancy in RET% between the instrument method and Faster R-CNN counting.

The upper limit of detection of RET% by the Sysmex XN instrument method is 30%, while the Faster R-CNN can count higher RET%. Brown and Wittwer (2000) found that the instrumental method to detect RET and its parameters were directly influenced by Howell-Jolly corpuscles, plasmodium, large platelets, red blood cell aggregation, cold agglutinin, or drugs. To avoid inaccurate test results caused by interference, it is routine practice at the PUMCH laboratory to transmit the RET% scatter plot to the computer of the verifier. The results can be double-checked and analyzed in combination with the scatter plot detected by the instrument, and only the samples with abnormal scatter plots need to be verified by manual microscope examination.

## 6 Limitation

This research has a limitation in that only the undisturbed sample were compared, and we did not select samples with interfering items for testing. Therefore, in future research, the Faster R-CNN needs to be used to detect samples containing interference items, calculate the RET%, confirm the effect of various interference factors on the detection of the RET% in the automated hematology analyzer, and ensure that the clinical laboratory can provide more accurate detection results.

## 7 Conclusion

In this study, the experimental results show that the Faster R-CNN can act as a rapid computer-aid tool for manual RET enumeration for cytological examiners. The Faster R-CNN analysis system provides better traceability, and the images captured using Faster R-CNN can be preserved permanently. The Faster R-CNN is faster than manual RET enumeration for cytological examiners.

## Data Availability

The original contributions presented in the study are included in the article/supplementary material, further inquiries can be directed to the corresponding author.

## Abbreviations

RET, reticulocytes; CV, coefficient of variation; RNA, ribonucleic acid; RET%, RET percentage; RET#, the absolute value of RET; IRF%, immature RET percentage; MFR%, low fluorescence RET ratio; RET%, medium fluorescence RET ratio; SOP, Standard Operating Procedure; SNCS, Sysmex Network Communication Systems; X-axis, the lateral fluorescence; Y-axis, forward scattered light signals; SD, standard deviation.

## References

[B1] Bain B. J. (2005). Diagnosis from the blood smear. N. Engl. J. Med. 353 (5), 498–507. 10.1056/NEJMra043442 16079373

[B3] Brown M. Wittwer C. (2000). Flow cytometry: principles and clinical applications in hematology. Clin. Chem. 46 (8 Pt 2), 1221–1229. 10.1093/clinchem/46.8.1221 10926916

[B4] Da Costa L. (2015). Digital image analysis of blood cells. Clin. Lab. Med. 35 (1), 105–122. 10.1016/j.cll.2014.10.005 25676375

[B5] Gu S. Wei G. (2013). Clinical application of reticulocyte parameter detection in diagnosis and treatment of iron deficiency anemia. Chin. J. Gerontology 33 (21), 5444–5445.

[B6] Health industry standard of the People's Republic of China (2012) WS/T 346-2011 Reference method for reticulocyte counting[S].

[B8] International Council for Standardization in Haematology, Writing Group Briggs C. Culp N. Davis B. d'Onofrio G. Zini G. Machin S. J. (2014). ICSH guidelines for the evaluation of blood cell analysers including those used for differential leucocyte and reticulocyte counting. Int. J. Lab. Hematol. 36 (6), 613–627. 10.1111/ijlh.12201 24666725

[B9] Liao H. Chen J. Mao Z. (2016). The establishment and application of intelligent management system for XN-9000 blood analysis pipeline. C hin J. Clin. Lab. Sci. 34 (5), 394–397. 10.13602/j.cnki.jcls.2016.05.22

[B10] Li L. Zhang L. Li W. (2014). Comparison of reticulocyte count results deteced by blood cell analyzer and manual method and its clinical significance. Int. J. Lab. Med. 35 (14), 1928–1929.

[B11] Liu Z. (2018). Application of reticulocyte and related parameters in diagnosis of anemia. Guide China Med. 16 (31), 137–138.

[B7] Luan H. Zheng J. Dong X. H. Zhou L. P. (2011). Clinical application of reticulocyte parameters in anaemia differential diagnosis. J. China Med. Univ. 40 (11), 1018–1019.

[B12] Mohammed E. A. Mohamed M. M. Far B. H. Naugler C. (2014). Peripheral blood smear image analysis: a comprehensive review. J. Pathol. Inf. 5 (1), 9. 10.4103/2153-3539.129442 PMC4023032 24843821

[B2] Peng B. Ma Y. N. He F. Qiu X. Q. Ou H. J. Ou Yang Q. H. (2018). Diagnostic value of reticulocyte related parameters in thalassemia and iron deficiency anemia. Int. J. Lab. Med. 39 (2), 153–155.

[B13] Wang G. Zhao T. Fang Z. Lian H. Wang X. Li Z. (2021). Experimental evaluation of deep learning method in reticulocyte enumeration in peripheral blood. Int. J. Lab. Hematol. 43 (4), 597–601. 10.1111/ijlh.13588 34014615

[B14] Wang J. J. Yu Y. L. Ren C. J. Liu X. (2013). The preliminary discuss of reticulocyte parameters in large cell anemia diagnosis. J. Clin. Transfus. Lab. Med. 15 (1), 39–41.

[B15] Wang K. (2010). An study on the principles of counting and classification of 5-part differential hematology analyzer. Med. Equip. 23 (1), 47–48.

[B16] Wu X. Zhang Q. (2012). Comparison of reticulocyte count with manual count by Sysmex XE-5000 automatic blood cell analyzer. Int. J. Lab. Med. 33 (16), 2013–2015.

[B17] Zhang W. Ding D. (2016). Application of reticulocyte and red blood cell related parameters in differentiation diagnosis of anemia. Int. J. Lab. Med. 37 (1), 65–66.

